# Quitline Queensland: the journey to a globally unique smoking cessation service in Australia

**DOI:** 10.3389/fpubh.2025.1576541

**Published:** 2025-07-08

**Authors:** Joanne Isbel, Madonna Kennedy, Mark West, Kerry-Ann F. O’Grady, Shelley Peardon-Freeman

**Affiliations:** ^1^Health Contact Centre, Clinical Excellence Division, Queensland Health, Herston, QLD, Australia; ^2^Prevention Strategy Branch, Population Health Division, Queensland Health, Herston, QLD, Australia; ^3^Centre for Healthcare Transformation, Australian Centre for Health Services Innovation, Queensland University of Technology, Kelvin Grove, QLD, Australia

**Keywords:** smoking cessation, Quitline, nicotine replacement therapy, counselling, Queensland (Australia)

## Abstract

Smoking cessation programs remain a core component of global efforts to reduce smoking and nicotine addiction. Telephone-based counselling, with or without the provision of nicotine replacement therapy, commonly referred to as Quitlines, has been a cornerstone of smoking cessation programs and they vary in scope and content and the populations they target. We describe the history, and structure of Quitline Queensland, Australia that was implemented in 1997. Quitline Queensland offers intensive quit support programs incorporating 4 weeks of telephone-based counselling, free nicotine replacement therapy mailed to participants and up to 12 months of follow-up. The program has evolved through a strong government commitment to, and support for, evidence-based solutions to reducing the burden of smoking in Queensland. Eligible cohorts have been identified by evidence-based reviews, equity considerations, trends in smoking prevalence and to address new challenges to smoking cessation such as vaping and the impact of the COVID-19 pandemic. New approaches to engaging and retaining smokers and delivering the program are being evaluated and implemented.

## Introduction

Tobacco smoking remains a leading cause of morbidity and mortality worldwide, including being the major attributable risk factor for disability-adjusted life years (DALYs) (1). Globally, health promotion, education, legislative restrictions and smoking cessation programs have become cornerstones of public health approaches to reducing the uptake and prevalence of smoking. In Australia, these combined measures have halved the prevalence of smoking since 1991 (2) and, in 2022–23, 8.8% of Australian adults overall were current smokers (3).

A core component of initiatives to reduce smoking prevalence has been evidence-based, telephone-based, client-centered counselling and coaching (4), with or without nicotine replacement therapy (NRT) and/or other pharmaceuticals such as varenicline and bupropion. Originating from a cancer information service in the United States (US) in the early 1980’s (5), in many countries, including Australia, these programs are commonly known as Quitline. These services range from one-off contacts with people calling in for ad-hoc advice through to multi-contact programs with several contacts at defined timepoints from the counsellors to those registered for ongoing support (6). Depending on jurisdiction, clients have initial contact through either self-referral or via healthcare professionals in different settings.

The provision of NRT may or may not be included and, if so, it ranges from free product through to subsidies to reduce the cost of NRT to the client for defined periods of time. Globally in 2019, less than a third of countries with smoking cessation programs either partially or fully covered the costs of NRT for people attempting to quit (7). The first Quitline service in Australia was established in the state of Victoria in 1985 (5) and the Australian Government has subsidised smoking cessation pharmaceuticals (including NRT) through the Pharmaceutical Benefits Scheme (PBS) since 2014.

As efforts continue to reduce smoking prevalence and new threats to smoking cessation emerge, for example vaping (8), the need for ongoing, comprehensive support to people who smoke remains a public health priority. With the aim of supporting the ongoing development and implementation of Quitline services internationally, here we describe the history of the Intensive Quit Support Program (IQSP) in Queensland, Australia, a unique Quitline program in the Australian context, and in many places globally, given its comprehensive model of multiple counselling sessions delivered by trained counsellors over the telephone in conjunction with provision of up to 12–16 weeks of free NRT. We describe the components and delivery of the IQSP in a demographically and geographically diverse population. The paper does not describe a formal evaluation of the service using an implementation science framework (9) rather it is an historical account of the development and components of the service over time.

## Context

### Setting and demographics

Queensland has the third largest population in Australia (5.2 million at the 2021 Census) (10) and the second largest in geographical size (1.730,648 km^2^) covering 23% of Australia’s landmass. Two-thirds of the population live in major cities, with the majority of those concentrated in the South-East corner of the state and approximately 2% live in remote and very remote communities (10). In 2021, the median age was 38 years, 22.7% were born overseas and 3.2% of the population identified as being of Aboriginal and/or Torres Strait Islander origin (10). The median weekly income for individuals in 2021 was AUD$787 and AUD$2024 for families and 38% of the population were in the two most disadvantaged socio-economic quintiles for Australia (10). Geographically, the state is diverse ranging from tropical rainforest in the North to dry arid desert in the South-West.

### Smoking prevalence

Daily smoking prevalence in Queensland has declined by 47% since 2002 and, in 2022, 10.4% of adults smoked daily (11); 4.7% smoked less often than daily, 28.0% were ex-smokers, and 56.9% had never smoked. The proportion of smokers who have quit smoking increased by 9.2% from 2009 to 2022 (from 58.5 to 65.0%). There was no evidence that quit rates varied by sociodemographic characteristics. However, daily smoking prevalence was 3-times higher in the most disadvantaged areas than the most advantaged and almost 80% higher in remote areas compared to major cities (11). For Australians overall, in 2022 8.3% of persons aged 14-years and older were daily smokers (8.8% of persons aged 18-years and older). When combining daily, weekly and less than weekly, the prevalence of smoking was 10.5% for persons aged 14-years and older and 11.1% for those aged 18-years and older (12).

The prevalence of current (i.e., daily + less often) smoking among Aboriginal and Torres Strait Islander peoples in Australia aged 15-years and older has declined from 54.5% in 1994 to 31.5% in 2022–23 (13). Declines occurred in all adult age groups except for adults aged 55-years and older. In 2022–23, more than two in five Aboriginal and Torres Strait Islander peoples aged 18- years and older who had previously smoked had quit (13). The prevalence of current smoking among Aboriginal and Torres Strait Islander adults is consistently higher in those living in remote areas compared to non-remote areas (57 vs. 27%, respectively, in 2022–23) (13).

In 2022, 8.3% of all Australian women who gave birth smoked at some time during pregnancy, down from 13.0% in 2011 (14); the corresponding proportion in Queensland was 11.4% in 2022, down from 16% in 2011. Nationally, in 2022, teenage mothers were almost 6-times more likely to smoke during pregnancy than mothers aged 35—39 years (29.3 vs. 5.1%) and 39.7% of Aboriginal and Torres Strait Islander women reported smoking during pregnancy (14). The prevalence of smoking at any time in pregnancy among Aboriginal and Torres Strait Islander mothers nationally was 39.7% (14).

In 2024, 25.9% of Queensland adults had ever used an e-cigarette and 7.7% currently vaped (15). In 2022–2023, 35.6% of Queensland high school students 12–17 years had ever used an e-cigarette and 21.3% had vaped in the previous month. The proportion of adults who currently use e-cigarettes increased by 4.0 times from 2018 to 2024, while the proportion who vape daily increased by 5.9 times in the same period (15). The proportion of Queensland high school students 12–17 years who had ever vaped doubled from 2017 to 2022–2023, and the proportion who had vaped in the past month more than tripled in the same period (15).

### Health services

Health services are provided by the public and private sectors with the public sector, Queensland Health, divided into 16 Hospital and Health Service (HHS) districts across the state (16). Queensland is also serviced by multiple private hospitals, non-government organizations, Aboriginal and Torres Strait Islander Community Controlled Health Organizations, general practices and seven primary health networks. Quitline services in the state are funded by Queensland Health and operated by the Health Contact Centre (HCC), a confidential health assessment and information service that operates 24 h a day, 7 days a week using multi-channel delivery models.

### Australian responses to smoking

Australia has been a global leader in its public health response to smoking (17) that has included increasing taxes, graphic health warnings on packages and plain packaging, mass media campaigns, smoke free places policies and harm reduction approaches. Queensland Health has been strongly supportive of all measures including high-level political support for reducing smoking including the introduction of the Tobacco and Other Smoking Products Act in 1998 (18) and further related legislation that has resulted in Queensland having some of the toughest anti-smoking laws globally (19). Queensland implemented its first Quitline service in Queensland in 1997 which has consisted of both single contacts in a comprehensive call quality framework and was followed by an intensive quit support program (IQSP) introduced in 2005. Individuals can seek support at any time with calls answered 24 h a day, 7 days a week.

## Programmatic elements

### The intensive quit support program Queensland timeline

The rapidly expanding legislative and other measures to reduce tobacco smoking necessitated the implementation of further measures to support attempts to quit. Amendments to Queensland’s Tobacco and Other Smoking Products Act in 2004 introduced what were at the time the most comprehensive smoke free laws in Australia, taking effect from 1 January 2005 (20). In 2005, Queensland Health implemented a new Smoking Management Policy that included the first IQSP in Queensland, at that time titled “*Quit Smoking…for life!*,” targeting staff of Queensland public hospitals who wanted to quit (21). *Quit Smoking….for life*! consisted of six support calls plus 16 weeks of free NRT and 1,000 staff members registered in the first month of operation. The program was part of a suite of related initiatives including the introduction of free NRT to inpatients of Queensland public hospitals, training of frontline hospital staff in the delivery of brief quit smoking interventions and the ban of smoking in all Queensland Health facilities, grounds and motor vehicles except for specifically nominated outdoor smoking places that took effect from 1 July 2006.

The scope of the IQSP progressively expanded over subsequent years (Table 1), with the number of counselling sessions ranging from 4 to 8 and between 12 and 16 weeks of NRT. Eligible cohorts have been identified and incorporated based on smoking prevalence, priority populations such as pregnant women and First Nations Australians and in response to acute public health events such as the COVID-19 pandemic and, more recently, the rapidly increasing prevalence of e-cigarettes (vapes) (11).

**Table 1:** Development of Queensland’s intensive quit support program.

Financial year (FY) commenced	Program name	Cohort and eligibility criteria	Status
2005–06 FY	Queensland Health Staff	Staff employed by Queensland Health	Ongoing
2011–12 FY	Workplace Quit Smoking Support (WQSP)	Organizations that employed blue collar workers	Finished June 2019
2014–15 FY	1. Corrective Services Staff 2. Smoke-Free Mums and Bubs Pilot Program	1. Staff of Queensland Prisons - coincided with commencement of Smoke-free Prisons 2. Pilot program for First Nations pregnant women and their partners	Finished June 2015 Finished June 2015
2015–16 FY	1. Queensland Ambulance Staff 2. Quit for you…Quit for Baby (QFYQFB)	1. Queensland Health staff program expanded to include Queensland Ambulance staff 2. All Pregnant women who smoke and want support to quit	Ongoing Ongoing
2016–17 FY	1. People experiencing disadvantage (Quit for You) 2. Quit for You…Quit for Baby	1. Individuals who are clients of organisations that support people experiencing socio-economic disadvantage 2. Expansion of the program to include partners of pregnant women	Ongoing Ongoing
2017–18 FY	1. Rural, regional, and remote (RR&R) 2. Yarn to Quit (First Nations clients) 3. Priority Groups	1. Residents of Queensland Hospital and Health Services that had an adult daily smoking rate ≥14% (n = 9) 2. First Nations residents of Queensland 3. Expanded to include clients of Queensland Health Community Mental Health services	Ongoing – eligibility amended in 2018–19 FY Ongoing Ongoing - Commenced November 2017
2018–19 FY	1. Staff and students of Technical and Further Education (TAFE) Colleges 2. Patient Wellness Program 3. Regional, Rural and Remote 4. Quit for You…Quit for Baby (QFYQFB)	1. Coincided with the implementation of TAFE campuses going smoke-free 2. Clients of the Patient Wellness Program 3. Residents of Queensland Hospital and Health Services that had an adult daily smoking rate ≥10% (n = 10) 4. Expanded to include individuals who were planning a pregnancy within the next 6 months	Commenced March 2019 Ongoing - name changed to Way to Wellness program. Ongoing – eligibility amended in 2019–20 FY Ongoing
2019–20 FY	1. Staff and students of Technical and Further Education (TAFE) Colleges 2. Smoke-free Families 3. Regional, Rural and Remote (RRR) 4. South-East QLD cohort 5. COVID-19 Hotel Quarantine	1. As above 3. Residents of Queensland Hospital and Health Services that had an adult daily smoking rate ≥12% (n = 11) 4. All residents of Queensland could access intensive quit support as a response to the Covid-19 pandemic 5. Individuals who were required to spend time in quarantine as a response to the Covid-19 pandemic. Clients received a 2-weeks supply of NRT and at least one contact session with Quitline	Finished September 2019 Ongoing Commenced April 2020 Commenced April 2020
2020-21FY	1. South-East QLD cohort 2. COVID-19 Hotel Quarantine	1. As above 2. As above	Finished August 2020 Finished August 2020
2021-22FY	No new cohorts		
2022-23FY	1. Priority groups	1. Expanded to included individuals due to be released from a Queensland prison Expanded to include clients from Queensland Alcohol and Other Drugs Services	Ongoing Ongoing
2023-24FY	1. Priority groups 2. Adolescents and Young Adults	1. Expanded to included individuals on a Queensland Health surgery waitlist Individuals aged <30 years	Ongoing Commenced January 2024
2024-25FY	1. Parents and careers of children attending a secondary public school 2. Education Queensland Staff	1. Coincides with the implementation of a pilot program to address smoking and/or vaping cessation among students (aged 12–18) enrolled in participating state secondary schools 2. Staff employed by Education Queensland	Commenced October 2024 Commenced October 2024

^*^Service calls are counselling calls and do not include evaluation calls post completion of counselling program.

Since inception, Quitline Queensland has evolved from a handful of staff delivering the service, largely via paper records and landline phones through to a large multi-disciplinary service with complex data and communication systems. The Quitline service has progressed with the implementation of contemporary customer relationship management (CRM) and telephony applications. Queensland Quitline provides a comprehensive training period for staff followed by consistent learning and development activities and quality assurance. The Health Contact Centre is a Quality Innovation Performance (QIP) (22) accredited organization. In 2010, an identified role for Aboriginal and/or Torres Strait Islander counsellors was created to provide clients with the option to speak directly to an Indigenous counsellor.

### IQSP implementation

Implementation of the ISQP over time has been informed and maintained through an ongoing and adaptive formal quality management and assurance framework (23). The framework has guided extensive clinical, operational and technical change across the life of the IQSP via evaluation of service outcome and performance data, technology road maps and engagement in both formal and informal clinical networks. These activities have been integral to the program being consistent with core implementation outcomes, particularly acceptability, adoption, feasibility, cost, penetration and sustainability (9). External accreditation is undertaken by Quality Improvement Performance Ltd. (22) in accordance with the Quality Improvement Council (QIC) Health and Community Service Standards (24) which encompass governance, management systems, consumer and community engagement, diversity and cultural appropriateness, and service delivery.

For almost all IQSP programs, participants can self-refer by contacting the Quit phone number or self-initiated completion of a registration form online or be referred by a third party, primarily healthcare practitioners. In 2017–18, financial incentives were paid to Public Dental Services across Queensland’s Hospital and Health Services to encourage referral with this initiative becoming part of the wider Queensland Smoking Cessation Clinical Pathway for patients in public hospital and public dental services. Referral is also mandatory under Australia’s Pharmaceutical Benefits Scheme (25) for subsidized prices for pharmaceuticals such as varenicline and bupropion after the first 4 weeks of therapy (25). Financial incentives were also offered to Community Mental Health and Mental Health inpatient facilities. The predominant form of contact with people throughout the program is by telephone call initiated by a Quitline counsellor. These can be interspersed with SMS messages alerting registrants/participants to a pending call and other activities such as distribution of videos to participants that share information, boost knowledge and confidence in the service and provide additional educational content to support quitting. Contact attempt limits were initially limited to a maximum of six attempts however this was later changed to three attempts following an internal review indicating most individuals answered within this range with limited additional benefit of further calls. This limit does not include manual reschedules at participant request.

From 2017 to 18, IQSP has included four counsellor-initiated telephone contacts over 6-weeks for counselling and support, and three evaluation calls at 3-, 6- and 12-months following completion of the initial 12 weeks (Table 2) (26). The calls are weighted to be more frequent earlier in the program to boost engagement and commitment and to provide opportunities to review the treatment plan and NRT requirements. However, as the service is tailored, clients can move and book their calls as suits and the delivery/timing of calls can be varied across the client group and could be stretched over a longer time-period.

**Table 2:** Structure of the current IQSP program, Queensland.

Registration	Program calls (4 contacts) over 6 weeks*				Evaluation calls (3 contacts) over 12 months		
Self-referred or third-party referral	AQPSC	SC2	SC3	SC4 (end of program)	EV1	EV2	EV3
	Day 1	+ 2 weeks	+ 1 week from SC2	+ 3 weeks from SC3	+ 3 months from SC4	+ 3 months from EV1	+ 6 months from EV2
	12 weeks of NRT if requested, sent in 4-week batches at AQPSC, SC3 and SC4. It is possible to place an order at SC2 depending on the client’s treatment plan						

^*^Subsequent contacts are automatically scheduled as per timeline at the time of each successful contact.

The evaluation phases are primarily short contacts to determine participants’ current smoking status. In September 2024, a new evaluation framework was implemented. This focuses on smoking/vaping cessation outcomes and measures client progress at 12-weeks post the initial Assessment and Quit Planning Support Call (AQPSC) and 6-months post evaluation one. In the immediate future Queensland Quitline is exploring alternative ways for clients to contact the service, including webchat and/or message-based services.

Clients are eligible for one IQSP program every 12-months, except for pregnant women who are eligible for two, based on when the AQPSC for each program was completed. Before starting a client on a new program, counsellors are required to consider what changes the client made in the previous program and what might be different about the new quit attempt (26). Should a client’s program be closed due to client request or becoming uncontactable, a client may reactivate the program within 3 months of the closure date. However if the initial AQPSC was not completed and the file was closed, if a client makes contact more than 3 months after the closure date, the client can be enrolled in a new program (26).

Participants are offered combination NRT (patches plus an oral form) based on evidence-based best practice (27). This includes double patching where suitable. Clients are offered higher dose patches and can nominate their preference to use gum, lozenges, or mouth spray/mist. Clients are provided with 4-weeks treatment at a time posted directly to them, and only one form of oral NRT per order. Some clients may preference mono therapy, either patches or an oral form. Lower dose NRT options are available for clients between 12 and 15 years of age who are under 45 kg (28).

### Participation

The number of referrals and subsequent successful contacts with Quitline Queensland staff has varied over time given changes in the number and scope of target groups eligible for IQSP and the funding arrangements in place. For the financial years 2016–17 to 2023–24, the average conversion percentage, that is the proportion of referrals with successful contact, varied by target cohort and year (Table 3). Conversion ranged from lows of approximately 30% in the Yarn to Quit program for Aboriginal and Torres Strait Islander Peoples to highs of 96% for the Smoke-free Families and Way to Wellness programs. Conversion for the largest cohort, those residing in regional, rural and remote communities ranged from 48 to 70%.

**Table 3:** Quitline referrals and the proportion resulting in successful contact with Quitline Queensland staff by target group, 2020–21 to 2023–24 financial years.

Financial year	Cohorts	Referrals			Participation			Conversion rate		
		Self	Third-party	Total	Self	Third party	Total	Self	Third party	Overall
2023–24	QH/QAS^a^	317	n/a^f^	317	242	n/a	242	76%	n/a	76%
	RRR^b^	1,412	2,752	4,164	1,181	988	2,169	84%	36%	52%
	Priority groups	106	1,603	1709	98	733	831	93%	46%	49%
	QFYQFB^c^	154	631	785	128	215	343	83%	34%	44%
	Yarn to quit	379	953	1,332	271	237	508	72%	25%	38%
	Smoke free families	185	58	243	174	49	223	94%	85%	92%
	WTW^d^	n/a	155	155	n/a	108	108	n/a	70%	70%
	People under 30	297	150	447	220	33	253	74%	22%	57%
22–23	QH/QAS	310	n/a	310	216	n/a	216	70%	n/a	70%
	RRR	1,376	2,470	3,846	1,157	756	1913	84%	31%	50%
	Priority groups	80	1,378	1,458	74	650	724	93%	47%	50%
	QFYQFB	117	779	896	91	273	364	78%	35%	41%
	Yarn to quit	324	910	1,234	247	196	443	76%	22%	36%
	Smoke free families	220	64	284	201	58	259	92%	91%	92%
	WTW	n/a	121	121	N/a	116	116	n/a	96%	96%
21–22	QH/QAS	69	n/a	69	53	n/a	54	78%	n/a	78%
	RRR	1,427	1,626	3,053	1,252	454	1706	88%	28%	56%
	Priority groups	47	1,192	1,239	43	588	631	92%	49%	51%
	QFYQFB	149	928	1,077	132	353	485	89%	38%	45%
	Yarn to quit	413	750	1,163	334	157	491	81%	21%	42%
	Smoke free families	241	47	288	232	43	275	96%	92%	96%
	WTW	n/a	102	102	n/a	100	100	N/a	98%	98%
2020–21	QH/QAS	19	n/a	19	14	n/a	14	74%	n/a	74%
	RRR	2,289	2,556	4,845	1884	824	2,708	82%	32%	56%
	Priority groups	7	1,216	1,223	5	689	694	71%	57%	57%
	QFYQFB	149	1,047	1,196	132	439	571	89%	42%	48%
	Yarn to quit	486	852	1,338	358	223	581	74%	26%	43%
	Smoke free families	375	153	527	353	135	488	94%	88%	93%
	WTW	n/a	59	59	N/a	57	57	n/a	97%	97%
	SEQLD^e^	2,112	2087	4,198	1,694	656	2,350	81%	31%	56%
2019–2020	QH/QAS	149	n/a	149	126	n/a	126	85%	n/a	85%
	RRR	2,530	2062	4,592	2,229	893	3,122	88%	43%	68%
	Priority groups	21	1,308	1,329	19	828	847	91%	63%	64%
	QFYQFB	147	805	952	131	356	487	89%	44%	51%
	Yarn to quit	435	410	845	354	207	561	81%	50%	66%
	Smoke free families	104	23	127	94	20	114	90%	87%	90%
	WTW	n/a	22	22	n/a	21	21	N/a	96%	96%
	SEQLD	461	167	628	388	69	457	84%	41%	73%
2018–2019	QH/QAS	235	n/a	235	200	n/a	200	85%	N/a	85%
	RRR	2,247	1985	4,232	2005	796	2,801	89%	40%	66%
	Priority groups	53	1,390	1,443	47	946	993	89%	68%	69%
	QFYQFB	40	334	374	32	161	193	80%	48%	52%
	Yarn to quit	340	757	1,097	278	229	507	82%	30%	46%
	WTW	n/a	9	9	n/a	8	8	n/a	89%	89%
2017–2018	QH/QAS	276	n/a	276	246	n/a	246	89%	n/a	89%
	RRR	2023	1,565	3,588	1789	743	2,532	88%	48%	71%
	Priority groups	2	1,206	1,208	2	834	836	100%	69%	69%
	QFYQFB	14	119	133	13	62	75	93%	52%	56%
	Yarn to quit	393	319	712	265	239	504	67%	74%	71%
2016–2017	QH/QAS	324	n/a	324	284	n/a	284	88%	n/a	88%
	RRR	364	397	761	318	167	485	87%	42%	64%
	Priority groups	n/a	156	156	n/a	117	117	n/a	75%	75%
	QFYQFB	19	40	59	8	23	31	42%	58%	53%

^a^QH/QAS, Queensland Health/Queensland Ambulance Service; ^b^RRR, Rural, remote regional; ^c^QFYQFB, Quit for You, Quit for Baby; ^d^WTW, Way to Wellness; ^e^SEQLD, South East Queensland; ^f^n/a, not applicable.

With the exception of the Smoke-free Families cohort, conversion has been consistently and substantially higher across years and cohorts for those who self-referred compared to third-party referrals (Table 3). This likely reflects higher self-motivation to quit than may be expressed to a health practitioner at the time a third-party referral occurred. Studies suggest that there are also differences in characteristics between those who are self-referred compared to those referred by third parties (29–33). For the latter, they are less ready to quit, have lower levels of education and income, cultural/ethnic/racial differences, are younger and less likely to engage if referred by a health service other than a medical practice. Further, while referral to Quitline is mandatory in Australia for subsidized prescription of pharmaceuticals such as varenicline and bupropion, it is up to the client to engage once contacted by Quitline staff. It is not known whether the prescriber confirms their client is participating.

Engaging participants in Quitline has not been limited to self- or third-party referrals. Various initiatives have been implemented, including the 10,000 Lives program launched in November 2017 that aims to reduce smoking in the region from 16.7 per cent to 9.5 per cent by 2030 (34). The program includes a broad range of initiatives including local promotion of existing Quitline resources and campaigns to community champions, general practices, local councils, businesses, and community services through local events, education sessions, in-services, newsletters, media and social media. Overall, 3,207 individuals were referred to Quitline during the 26-months-post-launch compared to 1,594 during 26-months-pre-launch period (35). The number of referred individuals who completed Quitline increased by 330.7% and quit smoking by 308.3% in the post-launch period (35). The increase was higher among those aged 45-years and older, females and Aboriginal and Torres Strait Islander peoples. The 10,000 Lives program is one example of Quitline Queensland’s consistent and strong engagement with stakeholders including promotional work and invitations to present on the service model.

### Sustainability and flexibility

Sustainability refers to the extent to which the program is maintained/institutionalized within Queensland Health’s ongoing operations (9). As illustrated in Table 1, the IQSP has been in place now for 20 years and continues to expand. Decisions for Quitline’s ongoing operations have been based on: regular reviews of the evidence; continuous identification and monitoring of cohorts in most need of tailored smoking cessation programs (based on regular monitoring of smoking prevalence), and; the need to address equity in disadvantaged or high-risk populations. This support has led to sustained flexibility in addressing new challenges to smoking cessation.

The ability of the program to adapt to new challenges has been demonstrated and, the flexibility of Quitlines globally that enable adaption to meet needs over time is a known strength of the model (36). During the COVID-19 pandemic the Queensland Chief Health Officer extended the IQSP to all residents of Queensland to support the increased focus on public and individual health concerns, especially respiratory health. This initiative received a significant uptake from the community. In 2023, with the support of the Queensland Legislative Assembly, the Health and Environment Committee held a public enquiry into reducing rates of e-cigarette use in Queensland. A report, *Vaping: An inquiry into reducing rates of e-cigarette use in Queensland*’ (37) was produced and included 14 recommendations including addressing cessation support to meet population needs. Consequently, from 1 January 2024, Queensland individuals aged less than 30 years who vaped or smoked tobacco became an eligible cohort to receive IQSP from Queensland’s Quitline service, as well as increased enforcement and legislation measures to address vaping.

### Challenges

As with many large public health initiatives, the program has faced numerous challenges over time. Earlier years of the Queensland program, and others, faced limitations in resources, technology and the evidence-base for various Quitline strategies and approaches was limited (5). Lack of benchmarking for core components of Quitlines including accessibility, quality and cost-efficiency (5) left gaps in decision-making that likely impacted on reach, uptake and retention in earlier cohorts. Competing public health priorities and rapid rises in the cost of healthcare delivery, particularly acute care, present ongoing challenges; as the prevalence of tobacco smoking, and burden of disease attributable to smoking decline, (38) shrinking target populations may make justification of funding more difficult.

The predominant challenge to Quitline Queensland is the reach of the program, that is the absolute number, proportion and representativeness of individuals who are willing to participate in the program (39), from reaching all smokers in Queensland to bridging the gap between the numbers of people who are referred to those who subsequently engage in the program. The limitations of the reach of Quitline programs are not unique to Queensland and present challenges for many services globally (40–42). While, to some degree, reach is a factor of the cost of Quitline programs (42), potentially innovative methods to improve awareness of, and engagement with, the IQSP programs are required. While, in Australia, Quitline information is included on cigarette packets and notices at points of tobacco and vaping sale, and advertisements regularly appear on televisions, digital media and other forms of print advertising, these do not provide detail of what the service provides. Lack of awareness of what Quitline entails is a known contributor to lack of uptake (43, 44) and mechanisms to improve the skills, knowledge and confidence of potential participants at time of referral and a greater understanding of Quitline at the community level, as demonstrated by the 10,000 Lives program (35), can enhance awareness. Cold-calling via telephone directories has demonstrated some success in improving reach and uptake (45, 46) however require substantial additional resources to implement and maintain.

Conversion to the program once referral has occurred is consistently lower for referrals from providers than self-referrals. Providers report several barriers to referring patients to quitlines (47, 48) including limited knowledge about services offered, time constraints, difficulties with the referral process, and lack of feedback between providers and the service. Recommendations to improve referral and uptake include more patient and provider education, more efficient referral processes, and increased bi-directional communication between providers and the Quitline (48). Electronic referrals from within integrated medical records at the time of consultation, that minimize provider workload, coupled with brief smoking cessation advice have been shown to improve referral rates and uptake of the programs (49, 50). Quitline Queensland has currently implemented sending a SMS link to a video explaining the program once a referral has been received and increased materials on the service’s Webpage to assist potential participants in their understanding of what is required.

Further, while there are targeted programs and Aboriginal and Torres Strait Islander counsellors, estimates suggest 1.3–1.6% of Aboriginal and Torres Strait Islander peoples who smoke are clients of Quitline services nationally (29). These estimates are not dissimilar to non-Indigenous clients however retention in the program is lower (29). While data suggest Aboriginal and Torres Strait Islander Peoples are motivated to quit and have had a least one quit attempt (51, 52), further efforts are needed to engage them to quit, particularly those that are culturally appropriate and readily accessible. Cultural safety, the confidence and capacity of healthcare providers to refer, the trust consumers have in the health service provider and the support of Aboriginal Community Controlled Health Organizations and trained Aboriginal and Torres Strait Islander Health Workers are all important mechanisms to support increased engagement with the service (44, 53, 54).

Retention of participants over time also requires sustained efforts and constant assessment of new approaches (31, 55–58). An internal review of the Rural, Regional and Remote IQSP, the largest of the targeted programs, for the period 2017–2020 indicated approximately 47% of those commencing the program completed the fourth counselling contact and 17% completed the 12-month evaluation call (Quitline Queensland, unpublished data). A recent qualitative analysis of conversations held with clients during Quitline Queensland contacts suggested educational opportunities regarding pharmacotherapy adherence and misconceptions can improve client confidence and smoking cessation outcomes (59). To meet the challenges of cohorts with more complex needs (mental health clients, pregnant persons, young people), additional training is provided to staff to ensure evidenced-based tailored support is provided to address these challenges and clinical supervisors are available to receive escalated calls or support service delivery.

## Discussion

The structure and breadth of Quitline services varies globally, necessarily, given the need to address local circumstances and the varying levels of financial and political commitment. Queensland’s Quitline is not dissimilar to other services however its position in a jurisdiction with extensive smoking legislation and health promotion activities and long-standing delivery of extended, free NRT to cohorts considered priorities for smoking cessation is relatively unique. Australia’s Pharmaceutical Benefits Scheme (PBS) currently supports monotherapy only (patches) so without the IQSP clients need to fund their own evidence-based NRT treatment which is an ongoing challenge for many clients.

The provision of free NRT together with behavioral interventions has been shown to improve engagement with the Quitline programs and increase the proportion of clients who successfully quit and remain abstinent, particularly in low socio-economic groups. In the 2022–23 financial year, the proportion of people participating in the Quitline Queensland IQSP reporting smoking cessation at the end of the fourth counselling contact was 57%, followed by 40, 30 and 22% of retained participants at the 3-, 6- and 12-month contacts, respectively. An evidence-based rapid review of Quitline programs (60) reported provision of NRT was the most important factor in adherence to full Quitline counselling programs. Facilitating access to NRT such as through mail-outs and the degree of adherence to free NRT use has been identified as a factor associated with quit success (61–63). However, challenges to engagement and smoking and vaping cessation remain, including the influence of the internet and social media (64–66), and concerns regarding the security and privacy of personal information in digital modes of smoking cessation programs (67–69).

Perhaps the most important enabler of the sustainability (i.e., the extent to which the program is maintained/institutionalized within Queensland Health’s ongoing operations) of the program has been the ongoing prioritization of smoking cessation in Queensland, with strong political support from government and executive support and ongoing funding from Queensland Health. There remains strong commitment to the continuation of Quitline Queensland given the challenge that tobacco smoking and vaping continue to present to individual and population health (70–72), and the need to implement and evaluate new mechanisms for engaging and retaining participants. The growth of digital interventions, including web-based program delivery, mobile applications and chatbots, are offering new ways to reach, engage and retain participants (73) with varying effectiveness in different populations (74–78).

There is an ongoing need for Quitlines to be flexible to what consumers need or want from the program, in particular across different demographics and socio-economic profiles as smoking and vaping prevalence changes over time and new technologies, such as artificial intelligence, and social media platforms expand. Conversational artificial intelligence (AI) is demonstrating early potential of increasing engagement in smoking cessation (79, 80) although larger studies with standardized approaches and outcomes are needed. Further, there are concerns surrounding the use of AI ranging from protection of privacy, algorithmic bias, exploitation by the tobacco industry, data quality and scalability (80, 81) that need to be monitored and addressed if they are to be both effective and efficient.

There is now a plethora of smoking cessation mobile applications available to support smoking cessation that can be used in conjunction with formal Quitline programs or on their own. However, evaluations have identified high rates of irrelevance, poor or no functionality, and turnover, with few providing evidence-based support for smoking cessation (82). The lack of high-quality evidence for the role of mobile phone applications in smoking cessation, is largely due to the heterogeneity of existing studies (83). Day-to-day support beyond the Quitline service through a mobile application addressing real-time identification and management of triggers to smoking demonstrated higher quit rates than formal support alone in a feasibility study and further studies are planned (84). A further study suggested that effectiveness was greater for an application that addressed acceptance of smoking triggers rather than avoidance (85). If relevant, smoking cessation support services need to support clients with choosing an application that meets their specific needs and has an evidence-base to its effectiveness.

The future of Quitline Queensland includes ongoing implementation and evaluation based on evidence-based interventions, implementation theories, models and frameworks (TMFs) and implementation strategies (86), together with existing quality improvement frameworks. New mechanisms for delivery of the counselling components are being investigated including online and in person face-to-face support either individually or in groups. Current research is investigating consumer and provider preferences for the content and delivery of Quitline. New processes for evaluating program outcomes supported by enhanced information technology systems are being established. This will support Quitline Queensland’s ability to continue adapt to and address new and ongoing challenges to reducing smoking and vaping prevalence in Queensland. Most importantly, the public health importance and economic value of Quitline Queensland, and elsewhere, need continuing support via high-quality evidence, advocacy and political will to achieve its goals.

## Data Availability

The data analyzed in this study is subject to the following licenses/restrictions: access to Queensland Health data requires a formal request to the Department. Requests to access these datasets should be directed to shelley.peardon@health.qld.gov.au.
